# Long-term effectiveness of irreversible electroporation in a murine model of colorectal liver metastasis

**DOI:** 10.1038/srep44821

**Published:** 2017-03-22

**Authors:** P. Sánchez-Velázquez, Q. Castellví, A. Villanueva, M. Iglesias, R. Quesada, C. Pañella, M. Cáceres, D. Dorcaratto, A. Andaluz, X. Moll, J. M. Burdío, L. Grande, A. Ivorra, F. Burdío

**Affiliations:** 1 Department of Surgery, Hospital del Mar, Passeig Marítim 25-29, 08003, Barcelona, Spain; 2 Department of Experimental and Health Sciences, Universitat Pompeu Fabra, Parc de Recerca Biomèdica de Barcelona, Barcelona, Spain; 3 Department of Information and Communication Technologies, Universitat Pompeu Fabra, Carrer Roc Boronat 138, 08018, Barcelona, Spain; 4 Translational Research Laboratory, Catalan Institute of Oncology (ICO), Bellvitge Biomedical Research Institute (IDIBELL), Av. de la Granvia de l′Hospitalet, 199-203, 08908 L’Hospitalet de Llobregat, Barcelona, Spain; 5 Departament of Pathology, Hospital del Mar, Passeig Marítim 25-29, 08003, Barcelona, Spain; 6 Universitat Autònoma de Barcelona, Plaça Cívica, s/n, 08193 Bellaterra, Barcelona, Spain; 7 Department of Animal Medicine and Surgery, Faculty of Veterinary Medicine, Autonomous University of Barcelona (U.A.B.), Plaza Cívica, s/n, 08193 Bellaterra, Barcelona, Spain; 8 Department of Electric Engineering and Communications, University of Zaragoza, Pedro Cerbuna, 12, 50018 Zaragoza, Spain; 9 Serra Húnter Fellow, Universitat Pompeu Fabra, Carrer Roc Boronat 138, 08018, Barcelona, Spain.

## Abstract

Irreversible electroporation (IRE) has recently gained in popularity as an ablative technique, however little is known about its oncological long-term outcomes. To determine the long-time survival of animals treated with a high dose of IRE and which histological changes it induces in tumoral tissue, IRE ablation was performed in forty-six athymic-nude mice with KM12C tumors implanted in the liver by applying electric current with different voltages (2000 V/cm, 1000 V/cm). The tumors were allowed to continue to grow until the animals reached the end-point criteria. Histology was harvested and the extent of tumor necrosis was semi-quantitatively assessed. IRE treatment with the 2000 V/cm protocol significantly prolonged median mouse survival from 74.3 ± 6.9 days in the sham group to 112.5 ± 15.2 days in the 2000 V/cm group. No differences were observed between the mean survival of the 1000 V/cm and the sham group (83.2 ± 16.4 days, p = 0.62). Histology revealed 63.05% ± 23.12 of tumor necrosis in animals of the 2000 V/cm group as compared to 17.50% ± 2.50 in the 1000 V/cm group and 25.6% ± 22.1 in the Sham group (p = 0.001). IRE prolonged the survival of animals treated with the highest electric field (2000 V/cm). The animals in this group showed significantly higher rate of tumoral necrosis.

Irreversible electroporation (IRE) has recently emerged as a reasonable alternative to multimodal ablative therapy regimens for liver cancer^1^. In contrast to other physical ablation modalities, such as radiofrequency (RFA) or microwave ablation (MWA), IRE has the distinct advantage of not causing thermal damage and is therefore not influenced by the so-called *heat sink effect* that appears in the vicinity of large vessels^2^. This means that IRE has the potential to prevent tumor recurrence close to the vessels, while sparing the extracellular matrix of the supportive connective tissue and adjacent vital structures^3^,^4^.

The results of tumor treatment in pre-clinical studies^5^ in small and large animal models^6^,^7^,^8^ have demonstrated its safety and feasibility. However, the animal models used in the literature were mostly given cutaneous or subcutaneous implanted tumors^9^, which are not suitable for analyzing the implications of IRE for widespread clinical use in deep-seated solid tumors. Guo *et al*.^6^ reported IRE in a hepatocellular carcinoma tumor model, but the maximum follow-up period was only 15 days.

In recent years, a number of clinical studies have addressed the advantages of IRE in tumor ablations next to the portal/hepatic veins, major bile ducts and large vessels, thus avoiding the technical limitations of the thermal modalities^10^,^11^,^12^,^13^,^14^. The first results of these studies show an enormous variability in terms of tumor recurrence rates, ranging from 0% to 50%, depending on the series^12^,^15^,^16^,^17^. In a recent publication Niessen *et al*.^18^ demonstrated a local recurrence rate of up to 75%, depending on the underlying tumor disease, in a cohort of 25 patients undergoing 48 ablations. In the subsequent regression analyses they concluded that a tumor volume > 5 cm^3^ and underlying disease type present in the patients were factors independently associated with early local recurrence^18^,^19^. Animal models are therefore needed to clarify the histological changes experienced in ablated tumors after a long follow-up. In clinical practice these changes are extremely difficult to assess, as recurrences are normally evaluated by imaging diagnostic with MRI or CT. These methods approximate the grade of response by assessing the regression of the tumor size but there is often a poorly delineated ablation zone caused by a periablational inflammatory tissue, which decreases the accuracy of the calculated volumes, especially on CT^20^.

We recently demonstrated that it is feasible and tolerable to use IRE to ablate large portions of the liver (up to 40%), although specific premedication therapy had to be previously administered^21^. In the present work we have performed massive ablations that encompass a large volume around the tumor nodule so that we can rule out treatment failure due to improperly ablated tumor margins.

The main goal of the present study was to evaluate the effectiveness of irreversible electroporation in a liver tumor model in terms of survival after a long-term follow-up. An additional aim was to assess the specific histological response of electroporated tumors at this point, by quantifying the grade of tumoral necrosis and the residual viable tumor.

## Materials and Methods

The animal research protocol was approved by the Ethics Committee for Animal Experimentation of the PRBB (Barcelona Biomedical Research Park) and by the Government of Catalonia’s Animal Care Committee (FBP-13-77-74) according to their guidelines. These guidelines follow Directive 2010/63/EU of the European Parliament and of the Council of 22 September 2010 on the protection of animals used for scientific purposes.

### Animal model and tumor implantation

Forty-six male athymic-nude mice (20–30 g, 6-weeks old) were provided by Harlan Laboratories (Indianapolis, IN, USA). The animals were maintained under standard conditions with a laboratory diet and water ad libitum.

In order to create a locally advanced animal tumor model, the poorly metastatic KM12C human colon cancer cell line was used. This cell line is a poorly differentiated adenocarcinoma, which has a low capacity to develop extrahepatic metastasis^22^. Tumors were subcutaneously implanted and passaged in donor nude mice and subsequently extracted and minced to prepare tumor fragments of 2 mm per side for the implantation.

As previously reported^21^, the animals were anesthetized with a mixture of isofluorane and inhaled oxygen and analgesia was provided with buprenorphine (0.05–0.1 mg/kg s.c.) and meloxicam (1–3 mg/kg s.c.). The tumor was stitched to the hepatic capsule of the medial lobe using a monofilament suture. After the procedure, 15 days were allowed for the tumor to grow to a size suitable for the IRE procedure.

### IRE treatment and Follow-up

IRE ablation of the liver tumor was performed in the IRE-treated groups under general anesthesia 15 days after liver tumor implantation. The mice were anesthetized as described above, and the abdomen was prepared for surgery. Repeat midline subxiphoid laparotomy was performed to expose the liver with the tumor. The tumor was measured with a vernier caliper and the tumor size was plotted using the maximal diameter (Dmax_0_, along the orientation bearing the largest tumor diameter). Special consideration was given to the presence of peritoneal or extrahepatic metastasis In case of appearance of peritoneal metastasis the animal would not be considered for ablation.

**Figure 1:**
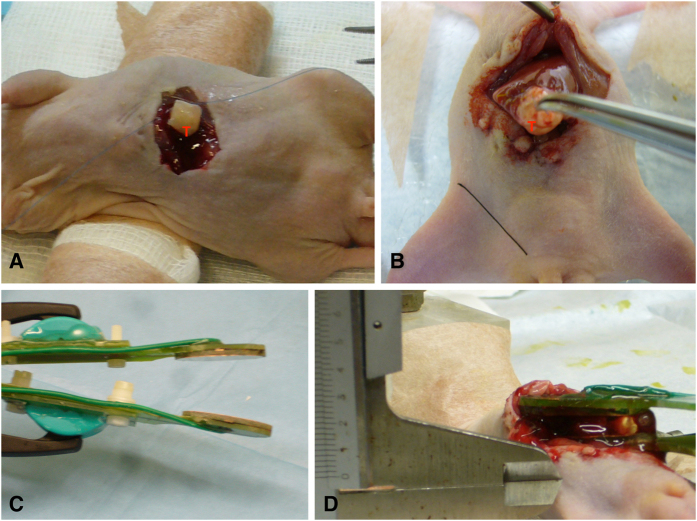
Setting for the procedure. (**A**) Representative images of tumor implantation in nude mice. (**B**) and (**D**) Scheme of IRE application procedure. (**C**) Photograph showing the plate electrodes used in this study.

After measuring the tumor, plate electrodes were placed as shown in Fig. 1D. In addition to the whole tumor volume between the two electrodes, both lobes of the left liver, which represents around 40% of total size^23^,^24^, were also included in the ablation zone. As previously published, a premedication was administered to prevent hyperkalemia and enable safe ablation of such a large volume of liver^21^.

A thin layer of partially conductive gel (Aquasonic 100 Sterile, Parker Laboratories, Fairfield, NJ, USA) was applied between the two parallel plate electrodes, as represented in Fig. 1C, to ensure a uniform electric field^25^. A custom made generator was programmed to deliver trains of ten pulses with a duration of 100 μs, a repetition frequency of 1 Hz and an electric field strength of 1000 V/cm or 2000 V/cm, according to the group. Ten of those pulse trains were applied (100 pulses in total). A pause of 10 s was performed between the pulse trains. The subset of sham operated animals were exposed to the conductive gel and positioned within the electrodes but were not electroporated.

The plate electrodes were ellipsoidal (maximum diameter = 25 mm, minimum diameter = 15 mm) and were made of gold coated copper on a custom made fiber glass printed circuit board (PCB) built using conventional PCB technology (Fig. 1C). The separation distance between the two plates ranged from 2 mm to 5 mm depending on the size of the tumor. Such separation was measured with a digital caliper in each animal before treatment. The voltage of the pulses was set so that the ratio voltage/distance matched the desired field (either 1000 V/cm or 2000 V/cm). During treatment, voltage and current were monitored for ensuring successful delivery with an oscilloscope (Agilent DSO1014A) through a high voltage probe (Tektronix P5100) and a current probe (Tektronix A622). The generator was recharged in between trains of pulses so that the voltage was kept constant during the whole treatment; relative voltage drop during each train of ten pulses was less than 10%. Currents of up to 20 A were recorded when pulses of 2000 V/cm were delivered.

The animals were returned to their cages for the duration of the follow-up and were checked daily to ensure that they had recovered, were healthy, and were not experiencing pain. Special attention was paid to skin ulceration, and the appearance of satellite lesions or a general decline in health suggestive of remote disease.

The principal end-point of the study was the overall survival (OS) and the effectiveness of the treatment measured by the percentage of necrosis achieved. The tumors were allowed to continue to grow in all the sub-sets until the animals met the end-point euthanasia criteria, given by the body condition score (BCS)^26^ and at this point the animal were euthanized and the tissue harvested for histology. The maximum follow-up time was 6 months. Those that survived longer were included as right-censored.

### Histology

Following the euthanasia, the tumors were measured (Dmax_1_) and photographed again. The liver was harvested immediately after sacrifice and fixed in 10% formalin. Fixed samples were embedded in paraffin, and sections of 3 μm were stained with hematoxylin and eosin (H&E) for microscopic analysis. The extent of tumor necrosis was semiquantitatively assessed and recorded as absent, focal (<10% of the tumor area), moderate (10%–30% of the tumor area) or extensive (>30% of the tumor area). Animals that worsened in BCS condition or needed to be sacrificed were also processed immediately after sacrifice.

### Statistical Analysis

A statistical analysis was performed using the SPSS statistical software package (SPSS, version 20, IBM, Armonk, NY, USA). Data is reported as mean ± standard deviation.

Increases in lesion size based on one-dimensional Dmax measurements were compared by a non-parametric Mann-Whitney U test. The survival curves (Kaplan–Meier curves) obtained were compared for the different treatments. A log-rank test was used to determine the statistical significance of the differences in time-to-event. The Cox regression model described how the hazard varies in response to IRE protocol. The results were expressed as Hazard Ratio (HR) with a 95% Confidence Interval (CI). Tests were considered statistically significant with a p-value < 0.05.

## Results

### Irreversible electroporation prolongs the overall survival of mice with liver tumors and reduces tumor growth rate

**Figure 2:**
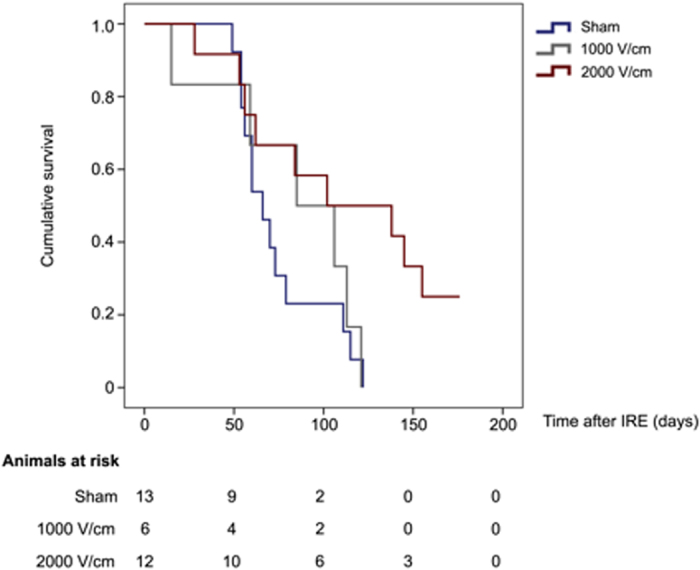
Kaplan-Meier analyses survival curve between the different groups. Mice treated with maximal voltage (2000 V/cm group) showed significant differences in survival as compared to untreated or suboptimally treated animals (Sham and 1000 V/cm groups) (Long-rank test <0.05).

A total of 46 animals were inoculated with the tumor, of which 3 did not develop parenquimatous tumors suitable for an IRE procedure. In the treatment group, 2 animals died during the procedure. One had a cardiac arrest before we began the IRE and other had a gas embolism. There were no deaths related to the placement of the electrodes, neither intraoperative bleeding as no needle electrodes were applied. The remaining 41 animals were distributed as follows: group 2000 V = 17; group 1000 V = 11; group Sham = 13. After the application of the pulses, there was a subset of 10 animals that survived less than 48 h (see Fig. 2). Beyond this group we attribute the early deaths directly to complications from the procedure or diselectrolytemia, so that they were excluded from the survival analyses.

We observed no differences between mean survival of the 1000 V/cm group and the sham group (83.2 ± 16.4 days vs 74.3 ± 6.9 days, p = 0.62). However, IRE treatment with the 2000 V/cm protocol significantly prolonged median mouse survival from 74.3 ± 6.9 days to 112.5 ± 15.2 days (see Fig. 2). A Cox regression analysis showed that the IRE-protocol applied was a variable independently associated with survival [HR = 0.55 (95% CI: 0.34–0.88; p = 0.011)].

Tumor size among the groups was comparable. The diameter of the implanted tumors at the time of the electroporation (Dmax_0_) presented no statistical differences among the groups (0.4 cm ± 0.05, p = 0.54). At the time of death, tumor size was significantly reduced (0.49 cm ± 0.1) for mice treated with maximal voltage (2000 V/cm group), whereas all the untreated or suboptimally treated animals (Sham and 1000 V/cm groups) showed increases (0.98 ± 0.1 cm) in the tumor measurement (p = 0.002).

### Histology

**Figure 3:**
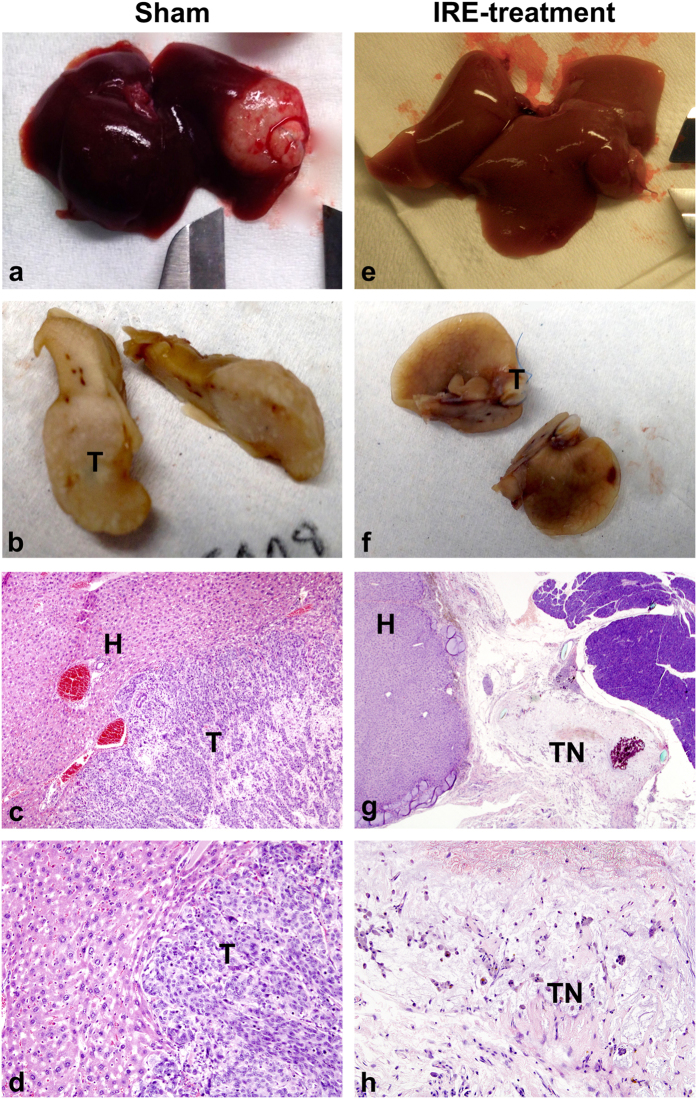
Gross pathologic sectioned specimen of the ablated murine liver compared with microscopic findings. Images of liver specimens harvested after autopsy (**a**) and after cutting and fixing in formalin (**b**) reveal the macroscopical appearance of the non-treated tumor compared to equivalent specimens of the 2000 V/cm treatment group (**e**) and (**f**). (**a**) Liver with large, whitish tumoral lesion. (**b**) Large white-tan tumoral lesion with small areas of necrosis and hemorrhage. (**e**) Liver with no macroscopic residual tumor. (**f**) Liver with small white area, without visual residual tumor. Microscopic observation of H&E preparations. (**c**) Interphase between structural healthy liver (H) and viable tumor (T) at bottom-right corner (4x’). (**d**) Detail of the interphase (10x) where the remaining tumor (T) reveals low percentage of intercellular necrosis. (**g**) Low magnification (2x’) of treated tumor after 176 days of survival, showing complete necrosis of the tumor (TN) with areas of inflammatory infiltrate and microcalcifications. (**h**) Detail (4x’) of the islands of lymphocytic infiltrates immersed in necrotic tissue.

**Figure 4:**
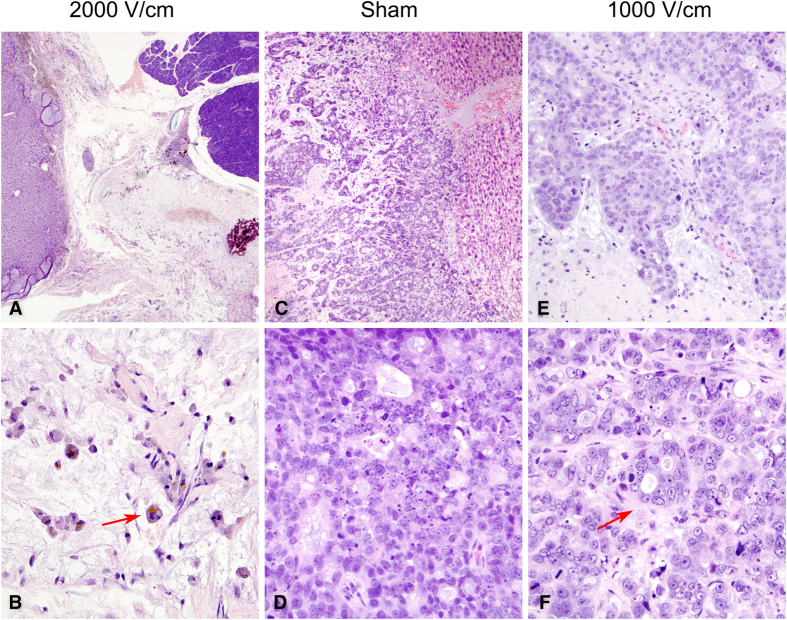
Haematoxylin and eosin (H&E) stained sections. H&E staining of the treated tumors from groups: 2000 V/cm (left column), sham (middle column) and 1000 V/cm (right column). (**A**) Extensive post-treatment necrosis of the tumor and preservation of the liver architecture (2x’). (**B**) Parenchymal shows only pale pink outlines of cell membranes with even paler cell contents, “ghost cells”, as a result of the treatment (20x’). Arrow shows inflammatory cells with remains of phagocyted material. (**C**) Poorly differentiated tumor of Sham group infiltrating the liver parenchyma (4x’) with very low percentage of tumoral necrosis (**D**) between the cells (20x’). (**E**) Animals from the 1000 V/cm group presented patchy areas of post-treatment necrosis (10x’) but significantly lower than the 2000 V/cm group (p = 0.001). However, in comparison with sham group, the representation of intercellular necrosis is higher in the 1000 V/cm group (arrow, **F**).

In the untreated Sham animals, tumors appeared macroscopically white and large (Fig. 3a and b) and in some cases with other organ involvement. Microscopically, the tumor appeared poorly differentiated with mucinous vacuoles in some cytoplasms, consistent with the initial diagnosis of adenocarcinoma. The cytoplasmic limits between the cells were barely distinguishable in several parts of the tumor and the nuclei showed athypia. Necrosis post-treatment was scarcely represented in this group. The mean percentage of tumoral necrosis of the Sham group was 25.6% ± 22.1 (Fig. 4D).

In the 2000 V/cm group, the treated lesions showed microscopically extensive necrotizing tissue, histocytes/lymphocytes reaction with residues of phagocyted material and microcalcifications. This subset of animals presented a higher percentage of tumoral necrosis post-treatment 63.05% ± 23.12 (p = 0.001), which was represented as large areas of cell ghosts (cells with pale pink outlines of membranes with dead nuclei) (Fig. 4B). This percentage of necrosis was significantly higher than in the Sham animals (p = 0.001). However, heterogeneity was observed in terms of tumor regression. In one of the animals there were no macroscopically or microscopically signs of remnant tumor, whereas the remaining 12 animals of the group showed incomplete regression but extensive tumor necrosis (>30%).

In the 1000 V/cm group the histological findings were similar to those of the Sham group. The tumors showed a moderate extent of necrosis (17.50% ± 2.50), but the percentage was not significantly different to that of the Sham group.

Six cases (46.7%) in the sham group and 6 animals in the 2000 V/cm group (31%) presented tumoral nodules in the laparotomy, which can be interpreted as distant metastasis. A Cox regression was performed to calculate the impact of these nodules on the overall survival. The crude hazard ratio (HR) was 3.02 (95%CI = 2.53–3.51, p = 0.004), which indicated a three times higher risk of death when the animals presented an implant in the laparotomy.

## Discussion

Irreversible electroporation is a promising technique and its advantages over other ablative techniques have been demonstrated in animal models and human clinical trials^4^,^12^,^14^. Recent studies show that despite an initial good response to the IRE treatment there is a high incidence of short-term recurrences^18^,^27^.

The initial results of on-going clinical trials in the literature reveal variable rates of response. Kingham *et al*.^12^ reported 5.7% of local recurrences in a retrospective cohort of 28 patients with a median follow-up of 6 months. Philips *et al*.^15^, with the largest series to date, describe a total of 31% local and distant recurrences evaluated by RECIST criteria. It should be noted, however, that that study includes an extremely heterogeneous sample of patients, mainly because of tumor characteristics, as 51.3% of these patients had large lesions with a high degree of vascular involvement. Also, the tumors were of different origin, which implies different patterns of recurrence and consequently an impact on the survival outcomes.

The series in Scheffer *et al*.^13^ and of Dollinger *et al*.^14^ included 10 and 43 patients, respectively, but did not specify whether the ablation was complete, nor did they provide clear results on survival. More recently, Niessen *et al*.^28^ described a rate of local recurrence of 25.2% in a serie of 34 patients in a 12-month follow-up.

As previously mentioned, most animal tumor models do not standardly evaluate the long-term outcomes, as animals are euthanized at most 3 weeks after treatment^6^,^29^,^30^. To the best of our knowledge, the present study is the first to evaluate the efficacy of IRE in an animal model with nearly 6 months of follow-up and provides evidence that IRE treatment with a high electric field strength reduces tumor growth and induces a larger percentage of tumor necrosis. In most clinical studies tumor response assessment is based on RECIST criteria, as no histopathological confirmation is routinely obtained^19^,^28^. A limitation of the animal study would be the lack of imaging monitoring. However, a decrease of the tumor mass by imaging does not necessarily imply changes in the cellular architecture or tumor death. Exclusive reliance on tumor size decrease does not provide a complete assessment of tumor response and may bring to erroneous conclusions. Our work shows for the first time in an animal model how the tumors treated with 2000 V/cm IRE suffer an extensive tumor regression with >30% of necrosis and animals potentially achieve a cure after at 6 months.

However, the most significant finding of the study is that a very high field is required to achieve a complete cure. Current IRE devices employed in clinical practice generate a voltage of between 1500–3000 V^12^ and create a target electric field of 680 V/cm, which is supposed to be the threshold for effective irreversible electroporation^31^. Although the electric field applied in the present study was notably higher and sufficient to theoretically ablate the tumor, some of the animals in the 2000 V/cm group still suffered a recurrence. The underlying cause of early recurrence still remains unclear. However, as a very large volume was involved, this study shows that recurrence is not due to failure to properly treat the tumor margins.

It seems reasonable that recurrence relies on insufficient or incomplete cellular ablation. In a recent work, Golberg *et al*.^27^ numerically and experimentally showed that the heterogeneity of the liver parenchyma affects the uniformity of the field distribution during IRE treatment and as a result the cellular death is uneven. Areas around the vessels in the plane perpendicular to the electrodes presented increased electric field strength, whereas the tissue surrounding the vessel on a parallel plane showed a marked decrease in electric field strength. The possible occurrence of this phenomenon had been identified previously by Ivorra *et al*.^32^ who hypothesized that the areas around the vessels could become sites of tumor recurrence.

From the foregoing it can be seen that the current electric field applied in clinical practice might not be sufficient to perform successful ablations due to the lack of homogeneity of the liver parenchyma. This could result in the under-treatment of the tumors and consequently play a role in early recurrence. Our results show that the group of 1000 V/cm achieved shorter survival and lower tumor regression. The greatest limitation of this study is the difficulty to compare these research outcomes with those from clinical practice as biophysical parameters concerning the strength of the electric field are often not accurate reported in the literature. Such heterogeneity in IRE algorithm applied in the different studies and the lack of standardization prevents drawing definitive conclusions. However, we consider the improved survival rate after a 6-month follow-up and the effective histophatological response in terms of tumoral necrosis to be encouraging results.

## Additional Information

**How to cite this article:** Sánchez-Velázquez, P. *et al*. Long-term effectiveness of irreversible electroporation in a murine model of colorectal liver metastasis. *Sci. Rep.* **7**, 44821; doi: 10.1038/srep44821 (2017).

**Publisher's note:** Springer Nature remains neutral with regard to jurisdictional claims in published maps and institutional affiliations.
